# Prolonged use of Kinesiotaping does not enhance functional performance and joint proprioception in healthy young males: Randomized controlled trial

**DOI:** 10.1590/bjpt-rbf.2014.0151

**Published:** 2016-03-18

**Authors:** Igor Magalhães, Martim Bottaro, João R. Freitas, Jake Carmo, João P. C. Matheus, Rodrigo L. Carregaro

**Affiliations:** 1 Faculdade de Educação Física, Universidade de Brasília (UnB), Brasilia, DF, Brazil; 2 Curso de Fisioterapia, Laboratório de Análise do Desempenho Funcional Humano, UnB, Campus UnB Ceilândia, Brasília, DF, Brazil; 3 Programa de Pós-graduação em Ciências da Reabilitação (PPG-CR), UnB, Campus UnB Ceilândia, Brasília, DF, Brazil

**Keywords:** physical therapy, athletic performance, postural balance, kinesiotaping

## Abstract

**Objectives:**

The aim of this study was to investigate the effects of continuous (48-hour) use of Kinesiotaping (KT) on functional and proprioceptive performance in healthy, physically active men.

**Method:**

Twenty-six healthy, physically active men (21.8±2.2 years old) were randomly allocated into two groups: 1) Kinesiotaping group (KG, tape applied with 40% tension for rectus femoris activation); 2) Control (CG, tape applied over rectus femoris without additional tension). Subjects attended the laboratory on five separate occasions: 1) familiarization; 2) baseline measurement without tape (BL); 3) immediately post-tape application (T0); 4) 24h (T24); and 5) 48h (T48) post-tape application. The outcomes were distance in the single (SHT) and triple hop tests (THT), vertical jump height (VJH), vertical jump power (VJP), and rate of force development (RFD). A mixed-model ANOVA was applied to verify differences between and within groups.

**Results:**

No significant (p >0.05) differences were found in the SHT and THT between groups and moments. Likewise, the main effects for VJH, VJP, and RFD were not significant (*p* >0.05).

**Conclusion:**

The present study demonstrated no significant immediate or prolonged (48h) effects of KT on functional and proprioceptive performance.

## Bullet points

- Previous studies assessed the immediate effects of KT on functional performance.
- KT applied with tension has no differences compared to a non-tension condition.
- The prolonged use of KT does not have a beneficial effect.
- KT is not recommended for functional performance enhancement in healthy subjects.

## Introduction

The Kinesiotaping (KT) method was created in the late 1970s and since then has been used widely in the sport and rehabilitation context^1^. The method is based on the application of an elastic adhesive tape that can be elongated up to 55-60% of its original resting length^2^ ^,^ ^3^ and can be used for several days. Recently, the KT method has been the focus of numerous studies on injury treatment^4^ ^-^ ^7^, proprioceptive support during joint movement^8^, and lymphatic circulation^9^.

This growing number of studies addressing KT is based on the proprioceptive and afferent stimuli of the elastic tape^10^ ^-^ ^15^. Recent findings demonstrated acute increases in eccentric muscle strength^13^, force perception^10^, and concentric elbow peak torque^16^. However, evidence regarding the effectiveness of KT during musculoskeletal rehabilitation is still inconsistent^1^ ^,^ ^2^ ^,^ ^17^. Furthermore, according to Martínez-Gramage et al.^18^, the evidence of the possible effects of prolonged use of KT on functional activities or human performance is still questionable and needs further clarification.

In this context, a valuable way to assess functional performance and rehabilitation effectiveness is through the hop and vertical jump tests^19^. The hop tests were described by Noyes et al.^20^ and have been used as a low-cost screening assessment^21^ of strength, power, proprioception, and neuromuscular performance. The vertical jump is a movement often used in sports and as a conditioning exercise to develop strength and power in the lower extremities^22^. Both movements consist of a multi-joint action involving the hip, knee, and ankle joints, with contraction of several muscles including the triceps surae, hamstrings, quadriceps, and lower back muscles.

There are contradicting results regarding the effectiveness of KT in hop tests and vertical jump performance, as previous studies found no acute significant effects^23^ ^,^ ^24^, while others confirmed some acute benefits of the KT^12^ ^,^ ^25^. In addition, there is a lack of studies on the prolonged effects of KT, as most studies focused on the acute responses^10^ ^,^ ^14^ ^,^ ^23^ ^,^ ^26^ ^,^ ^27^. This is noteworthy and conflicting, considering that the recommendation for the KT method is to use the tape for more than 24 hours in order to obtain the claimed effects. In fact, few studies compared the effects of KT on electromyography activity after 48h^28^ and 72h^18^, pain and disability within 48h of KT application for chronic low back pain^29^, and pain-free active range of motion scores within 1 day of KT use^5^. Thus, it is possible to assume that the increased peripheral nerve stimulation and recruitment of motor units attributed to the KT method may reach its maximal efficacy after 24h, and this could influence the performance of clinical assessments such as hop and vertical jump tests. This is in line with Vercelli et al.^26^, who recognized the need to investigate the effects on a prolonged application of KT. Therefore, it is hypothesized that the effects on a prolonged application of KT could increase muscle efficiency and, consequently, improve the performance of the hop and vertical jump tests. The aim of the present study was to investigate the effects of prolonged and continuous (48h) use of KT on functional and proprioceptive performance in healthy, physically active men.

## Method

### Study design

**Figure 1:**
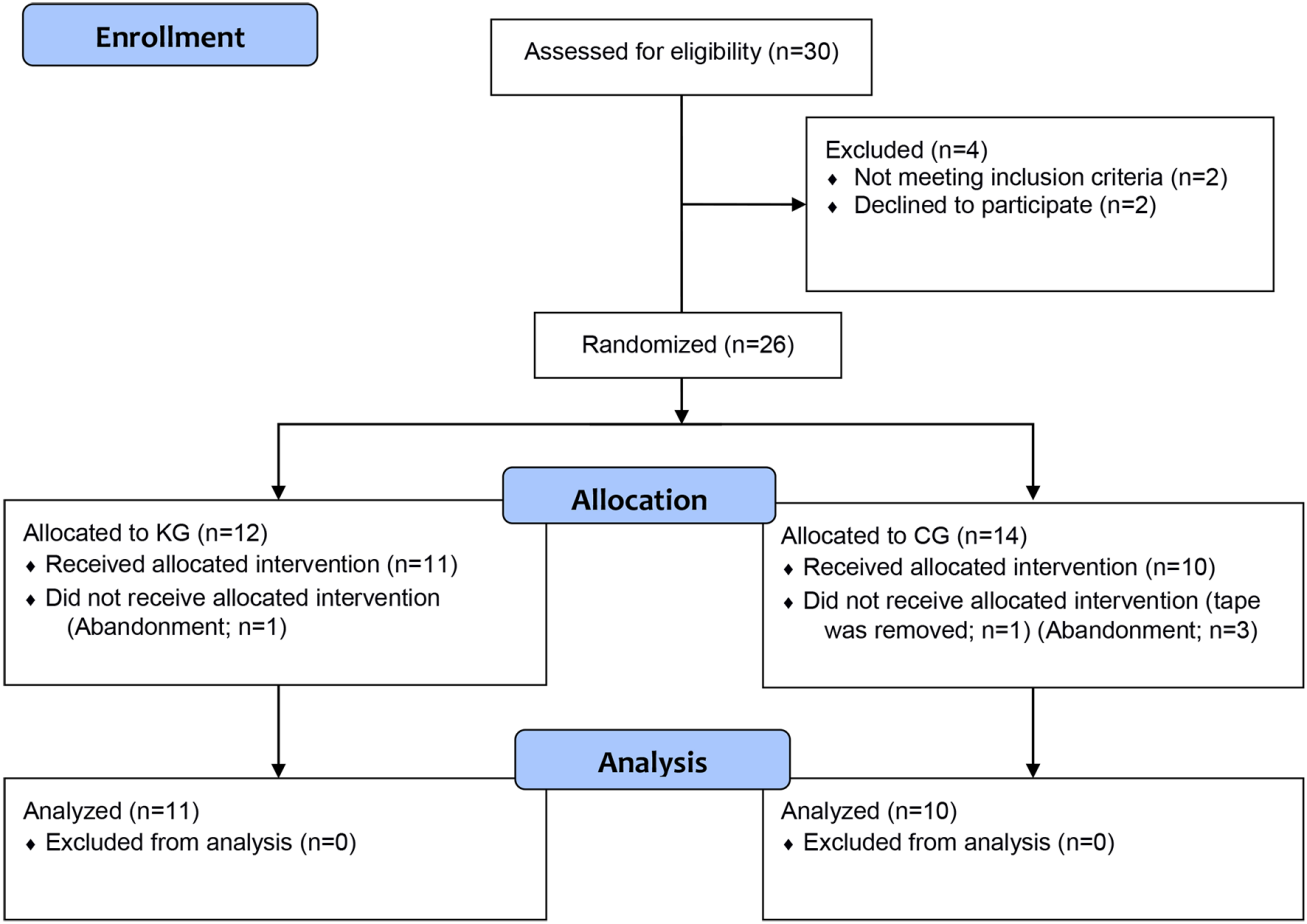
Study flowchart.

This is a randomized controlled trial (RCT) in which healthy, physically active men were randomly assigned to one of two intervention groups (Figure 1). This RCT was reported following the recommendations of the CONSORT Statement^30^. In addition, according to Miller et al.^31^, a deceptive design was adopted.

### Participants

Thirty healthy male subjects were selected at random from the respondents to fliers distributed to health sports clubs, posters placed in strategic points on the university campus, and by word-of-mouth. Sample size was calculated a priori using GPower software, version 3.1.9. Considering a statistical power of 80%, α-value of 5%, and a moderate effect size (*d* =0.5) between KT and control groups, a minimum of 22 subjects should be included in the study design.

Anthropometric data and physical evaluations were taken prior to the randomization procedure. The International Physical Activity Questionnaire (IPAQ)^32^ was applied in order to evaluate the physical activity level of the participants. Inclusion criteria were: a) males; b) aged between 18 and 30 years; c) physically active (classified as moderate or higher, according to the IPAQ questionnaire); d) height between 1.65 m and 1.85 m (in order to prevent anthropometric variability between subjects); and e) absence of pain and musculoskeletal symptoms. Exclusion criteria included open wounds or scars in the region of tape application, hypersensitivity, and erythema or lower limb injury in the past 6 months prior to the study. Participants who met the inclusion criteria were invited to read and sign an informed consent. The study was approved by the Institutional Research Ethics Committee of Faculdade de Saúde, Universidade de Brasília (UnB), Brasília, DF, Brazil (protocol n. 11350813.2.0000.0030).

Subjects who were selected to participate were admitted sequentially and randomly allocated to one of two groups: 1) Kinesiotaping group (KG, tape applied with 40% tension for rectus femoris muscle activation), and 2) Control Group (CG, tape applied without tension on the rectus femoris). For the randomization process, sequentially numbered sealed opaque envelopes containing the name of the intervention groups were used. Randomization was based on a table of random numbers generated by the website Random.Org^33^. This procedure was performed by an investigator who was blinded to the objectives and purposes of the study.

### Kinesiotaping application

For the present study, the Kinesio Tex Gold® tape (Albuquerque, NM, USA) was used and applied on clean and dry skin. For the KG, a tension of 40% was applied on the dominant limb (leg used to kick a ball), from origin to insertion (proximal to distal) according to the technique proposed by Kase et al.^3^ to facilitate the rectus femoris muscle activation.

Before the tape application, the subject lied in a supine position on a bench. Subsequently, the distance (DIS) between 10 cm below the anterior superior iliac spine (ASIS) and the tibial tuberosity was measured. In order to standardize and control the tape tension, after measurement, the strip was cut based on the Equation 1:

$$SB=\frac{\left(DIS-FP\right)}{1.4}+10cm$$ (1)

where:

*SB*: the size of the tape to be cut (cm);

*DIS*: distance between the point 10 cm below the ASIS and the tuberosity of the tibia;

*FP*: 10 cm of the anchors (5 cm each).

**Figure 2:**
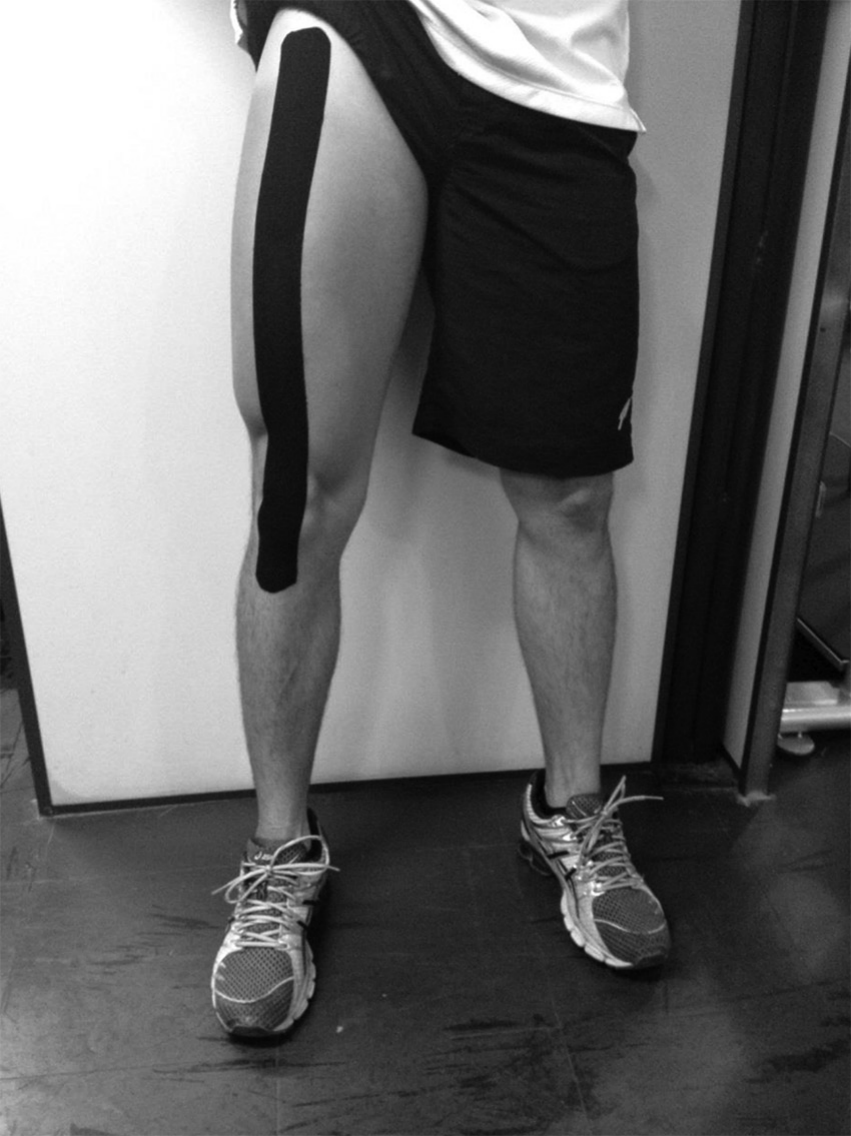
Illustration of the tape, after application.

After the calculation of the SB value and removal of the paper backing from the tape, the strip was applied with the subject lying on a bench with the leg positioned off, and the knee from the dominant limb flexed at 90º (Figure 2). The strip was stretched until it reached the DIS value, which according to the equation would produce a tension of approximately 40%. Tape was always administered by the same certified physical therapist (certified KT1/KT2). The CG used the same application and technique, however, no tension was added to the tape along the longitudinal line on the anterior thigh until reaching the tibial tuberosity. For the CG group, the equation was not applied. All participants received verbal and written guidance regarding tape care, diet, and exercise procedures during the study period. The subjects were also instructed to keep the tape on for 48h after the application.

### Testing procedures

After the process of randomization and allocation to the respective groups (KG or CG), the subjects attended the laboratory on five different occasions at 24h intervals: 1) familiarization; 2) baseline measurement (BL); 3) immediate post-application (T0); 4) 24h post-application (T24); and 5) 48h post-application (T48). The BL measurements were applied without tape, for both groups. After the tape was applied, subjects were instructed not to remove it. The testing procedures were applied and controlled by the same investigator, who was not blinded to the treatment allocation.

### Hop Tests

Two types of hop tests were used in order to assess the functional and proprioceptive performance^21^: 1) the single hop test (SHT) and 2) the triple hop test (THT). According to Ross et al.^34^, both tests present a high level of reliability (Intraclass Correlation Coefficient - ICC of 0.92 and 0.97, respectively).

**Figure 3:**
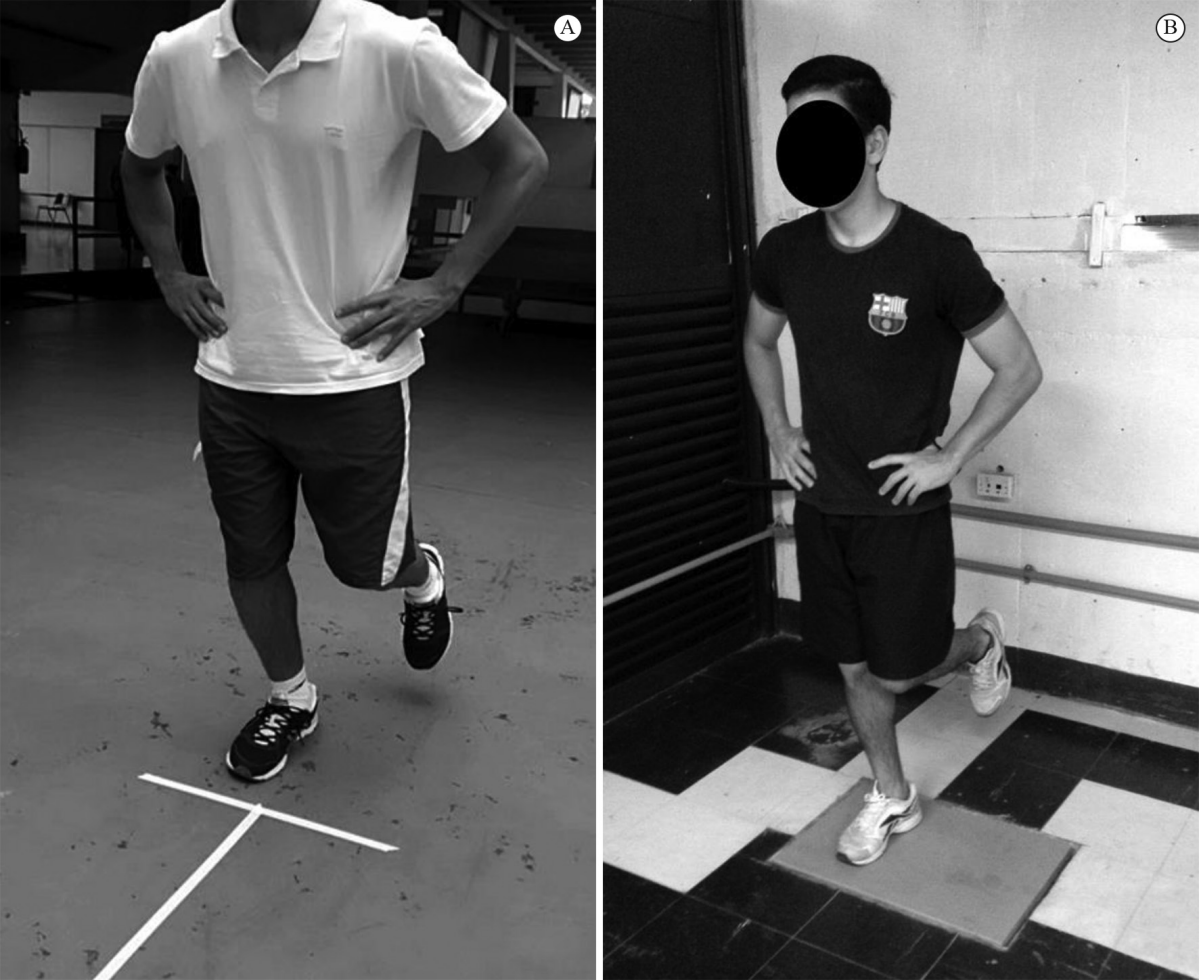
Illustration of the initial position of the hop test (A) and vertical jump test (B).

All measurements were taken from the dominant limb. The SHT started with the participants in single-leg standing behind a line marked on the floor with a knee slightly flexed for 10 seconds, until a verbal command was given (Figure 3). Immediately after the verbal command, they were asked to jump forward as quickly and as long as possible and to land with the same limb. In order to prevent influences of the upper limbs during the propulsion phase, subjects were instructed to maintain their hands on the waist. The jump was considered valid if the participants could maintain their balance for at least 5 seconds after landing. Subjects performed three SHTs with a 1-minute interval between tests, and the best attempt was used for analysis purposes.

The THT was performed with the same initial position. After the verbal command, subjects had to perform three consecutive and uninterrupted jumps as long as possible with the same limb on a straight line. As for the SHT, the attempt was considered valid if the participants could maintain the balance for at least 5 seconds after the third landing. Subjects performed three THTs with a 1-minute interval between jumps, and the best attempt was used for analysis. All subjects performed a 5-min warm-up walk before the tests. Subjects had a three-minute rest interval between the SHT and THT. Jump distance was marked on a measuring tape positioned on the ground for both tests.

### One-legged vertical jump

A force plate (AMTI, model BP400600-HF-2000; Advanced Mechanical Technology, Inc., Watertown, MA, USA) fixed at ground level was used to evaluate the jump height, jump power, and the rate of force development (RFD) during a one-legged vertical jump. The sampling rate was 1000 Hz. This test presents high levels of reliability for functional and strength performance (ICC: 0.94)^35^.

Following a 10-min interval after the hop tests, subjects were placed in front of the force plate. Initially, subjects had to step up on the platform and maintain a static one-legged upright position with their hands on the waist for 5 seconds, until a verbal command was given (Figure 3). After the verbal command, subjects were instructed to jump vertically as high as possible and land on the same limb. A 1-min rest interval was used between trials. Three trials were performed, and the best jump was used for analysis.

Data from the force plate software were exported to a text file (.txt) and analyzed in a Matlab subroutine (version 7.13 release 2011b, MathWorks Inc., Natick, MA, USA). The velocity curve was obtained by dividing the resultant ground reaction force by the subjects’ body mass, and the displacement curve was obtained by integrating the velocity signal. Finally, the displacement curve was integrated, in order to obtain the center of mass displacement at each instant. Thus, the greatest vertical displacement was considered as jump height (measured in cm)^36^ ^,^ ^37^. The RFD was calculated using the moment-time curve (0-30 ms interval) from the beginning of the acceleration phase of the jump^37^. Jump Power was obtained by multiplying the ground reaction force by velocity at the beginning of the jump^37^. Data were low-pass filtered (Butterworth 4^th^ order) with a cutoff frequency of 200 Hz.

### Statistical analysis

The Statistical Package for the Social Sciences (SPSS version 22.0) was used. Normality assumptions were confirmed by the Kolmogorov-Smirnov test, and data are presented as mean and standard deviation. The independent variable was tape condition (KG or CG). Dependent variables were SHT and THT distance (in cm), jump height (in cm), power (W/kg), and RFD (in N/s). The Box’s M test was used to verify the equality of covariance matrices. A mixed-model 2 X 4 ANOVA was used to verify differences between groups (KG and CG) and within moments (BL, T0, T24, and T48), with syntax according to the multivariate model. The effect size (ES) was calculated using the Cohen's *d* ^38^. The magnitude of the effect size was classified as small (d<0.50), moderate (d≥0.50) or large (d>0.8). Significance was set at 5% (*p* <0.05).

## Results

Thirty individuals were assessed for eligibility and included for enrollment in this study. Four participants were excluded for not meeting the inclusion criteria (2) and declined to participate (2) – Figure 1. During the intervention, four participants from the CG group and one from the KG were excluded due to withdrawal from the study or removal of the tape (Figure 1). The remaining 21 participants received the original assigned interventions and were included in the subsequent analyses. Demographic characteristics of the participants are presented in Table 1.

**Table 1:** Participants’ physical characteristics. Data are presented as mean (standard deviation).

	**KG**	**CG**
**Age (yrs)**	20.91 (2.23)	21.80 (2.22)
**Weight (Kg)**	78.78 (15.06)	83.17 (9.56)
**Height (m)**	1.74 (0.06)	1.78 (0.04)
**BMI (Kg/m^2^)**	25.97 (5.48)	26.23 (2.48)

BMI: Body Mass Index; KG: Kinesiotaping group; CG: Control group.

Data regarding the distance of the SHT and THT are presented in Table 2. For the SHT, no significance differences or interactions were found between groups (*F* =0.10; *p* =0.75) and moments (*F* =0.23; *p* =0.87). The THT presented neither significant differences between groups (*F* =0.97; *p* =0.33) nor moments (*F* =0.38; *p* =0.76). Small effect sizes were found for all comparisons.

**Table 2:** Values of the single hop test (SHT), triple hop test (THT), vertical jump height, power, and rate of force development (RFD) for the Kinesiotaping group (KG) and control group (CG). Data are presented as mean (standard deviation).

**SHT (% of body height)**										
**Group**	**BL**	**T0**	**MD [95% CI]**	**ES**	**T24**	**MD [95% CI]**	**ES**	**T48**	**MD [95% CI]**	**ES**
			**BLxT0**	**BLxT0**		**BLxT24**	**BLxT24**		**BLxT48**	**BLxT48**
KG	99.6 (11.9)	101.6 (12.4)	–2.0 [–7.5; 3.4]	0.2	100.2 (12.0)	–0.6 [–6.8; 5.5]	0.0	100.5 (11.6)	–0.9 [–7.5; 5.5]	0.1
CG	98.7 (10.6)	96.8 (12.3)	1.8 [–4.9; 8.7]	0.2	99.4 (10.0)	–0.6 [–6.5; 5.3]	0.1	98.4 (9.0)	0.3 [–5.9; 6.6]	0.0
**THT (% of body height)**										
KG	282.1 (44.2)	278.1 (44.2)	3.9 [–10.6; 18.5]	0.1	276.7 (46.8)	5.4 [–8.2; 19.1]	0.1	285.2 (47.7)	–.0 [–13.1; 6.9]	0.1
CG	283.5 (37.0)	290.6 (38.5)	–7.0 [–22.0; 8.0]	0.2	295.6 (32.2)	–12.0 [-29.8; 5.7]	0.3	287.8 (38.5)	–4.2 [–20.9; 12.4]	0.1
**Jump Height (% of body height)**										
KG	4.5 (0.8)	4.1 (0.5)	0.3 [–0.7; 1.4]	0.5	3.9 (0.8)	0.6 [–0.6; 1.9]	0.7	4.3 (0.8)	0.2 [–1.0; 1.4]	0.2
CG	4.8 (2.0)	4.7 (1.8)	0.6 [–1.5; 1.6]	0.0	4.8 (1.7)	0.01 [–1.5; 1.5]	0.0	4.3 (1.2)	0.4 [–1.7; 2.6]	0.2
**RFD (N/s/Kg)**										
KG	0.04 (0.03)	0.06 (0.03)	–0.02 [–0.07; 0.03]	0.7	0.06 (0.03)	–0.02 [–0.07; 0.02]	0.7	0.05 (0.03)	–0.01 [–0.05; 0.03]	0.3
CG	0.05 (0.03)	0.07 (0.05)	–0.02 [–0.1; 0.06]	0.7	0.06 (0.07)	–0.01 [–0.12; 0.09]	0.3	0.08 (0.09)	–0.02 [–0.17; 0.12]	1.0
**Jump Power (W/Kg)**										
KG	615.6 (169.3)	615.5 (128.6)	0.06 [–108.4; 108.5]	0.0	610.4 (142.6)	5.1 [–106.3; 116.6]	0.0	609.1 (126.7)	6.4 [–90.8; 103.7]	0.0
CG	626.5 (76.4)	664.1 (88.8)	–37.5 [–135.4; 60.4]	0.5	662.7 (66.7)	–36.1 [–160.4; 88.1]	0.5	627.7 (99.3)	–1.1 [–122.8; 120.5]	0.0

MD: Mean Difference; 95% CI: 95% Confidence Interval; ES: Effect size; BL: Baseline; T0: Immediate post-tape application; T24: 24h post-tape application; T48: 48h post-tape application; SHT, THT and Jump height were normalized by subject's height. RFD was normalized by subject's mass.

Vertical jump data are presented in Table 2. Jump height presented no significant differences between groups (*F* =0.60; *p* =0.44) and moments (*F* =0.75; *p* =0.46). Small effect sizes were found for all comparisons. Likewise, no significant differences were found on RFD between groups (*F* =0.04; *p* =0.83) and moments (*F* =0.48; *p* =0.69). Similarly, regarding the jump power no interactions were found between groups (*F* =0.34; *p* =0.56) and moments (*F* =0.57; *p* =0.63). Higher jump power values with moderate effect size were found for the CG group at T0xBL. However, for T48xBL a decrease on power performance and a medium effect size was also found (Table 2). For the KG, small effect sizes were found for all comparisons.

Concerning within-group differences, we observed that both the KG and CG group presented an increase in RFD with moderate effect size at T0 and the CG a large effect size at 48 h.

## Discussion

The present study evaluated the influence of Kinesiotaping applied to the rectus femoris muscle on lower-body functional and proprioceptive performance. The general findings demonstrated that the use of KT does not have a beneficial effect on functional performance of healthy, physically active individuals immediately after and up to 48h after post-tape application.

Regarding single and triple hop tests, the present study demonstrated no significant KT effects between groups or within moments. Recent studies found similar results on hop tests performance^23^ ^,^ ^26^. Lins et al.^23^ performed a randomized trial in which they compared the acute (immediate) application of three taping conditions. The KT applied over the vastus medialis and rectus femoris for quadriceps activation was compared with a group using tape without elastic properties, and a group without taping. The results demonstrated no effects from the KT and no significant differences between groups. The study of Vercelli et al.^26^ evaluated the acute application of KT on subjects of both sexes, and also found no significant KT influences on hop tests performance, corroborating our immediate post-tape application (T0) results. However, Aktas and Baltaci^25^ found significant acute effects of the KT on hop test performance. They evaluated healthy individuals of both sexes comparing four conditions: 1) control (no tape), 2) knee brace, 3) KT, and 4) KT plus knee brace. Similar to the present study, all subjects performed the single hop test and a vertical jump. The authors found a significant difference between control and KT application for male subjects, meaning that KT improved the jump distance during the single hop test. Regarding the vertical jump performance, the authors did not find any significant effects for all groups.

The improvement on hop test performance found in the study of Aktas and Baltaci^25^ was explained by underlying mechanisms claimed by the KT method and commonly described in the literature involving the method^39^. It was hypothesized by Aktas and Baltaci^25^ that the stimuli provided by the KT enhanced the proprioception by mechanical stimuli on muscular and joint peripheral receptors transmitted along afferent pathways of the sensorimotor system. According to Mandelbaum et al.^40^, these stimuli are crucial to neuromuscular control and motor performance. In addition, the KT method is purported to facilitate the effect of cutaneous mechanoreceptors, which would improve neuronal excitability and, consequently, muscle function^14^. Another mechanism claimed by the KT is a facilitatory effect of cutaneous mechanoreceptors that improves neuronal excitability and, consequently, muscle function^14^. However, Halseth et al.^41^ evaluated the effects of taping the anterior and lateral portion of the ankle as a strategy to enhance ankle proprioception compared to a condition without taping. Their findings demonstrated no proprioceptive enhancement of KT during a joint position sense task. Similarly, our study provides evidence that KT has no proprioceptive and performance enhancement from acute or prolonged application, contradicting the influences of the aforementioned KT mechanisms. It seems that for optimal improvement in sprint, jumping, and strength performance, resistance or plyometric training appears to be more effective^42^. For example, a previous study demonstrated significant effects of plyometric training on shoulder position sense, which was explained by peripheral adaptations resulting from repetitive stimulation of the articular mechanoreceptors near the end range of motion of the shoulder during the exercises^43^.

To the best of our knowledge, this is one of the few randomized trials that investigated the prolonged (48 h) effects of KT on functional performance, and the results did not support the hypotheses that the prolonged use of KT would be beneficial. In addition, the small effect sizes found for the SHT and THT reinforce the interpretation that prolonged use of KT is not effective. It was expected that the continued use of the KT could increase the afferent stimuli of the mechanoreceptors claimed by previous studies and, consequently, improve the proprioceptive responses and functional performance. However, as the functional performance is related to muscle strength^21^, our findings may be explained by the fact that KT has no effects on muscle strength^26^ ^,^ ^44^. It is possible that the effects of the KT application are more evident in different muscle groups and in subjects with musculoskeletal dysfunction. Previous studies^5^ ^,^ ^6^ demonstrated pain reduction in patients with neck dysfunction immediately and 24h post-tape application and an improvement on shoulder range of motion immediately post-application. In addition, Hsu et al.^45^ observed a significant improvement of the ascendant trapezius strength, when the KT was compared to a placebo condition in baseball players with shoulder impingement syndrome. Thus, further high quality randomized trials focusing on musculoskeletal dysfunctions and functional performance are recommended.

The present study found no significant influences of KT on vertical jump height, RFD, and power, corroborating Nakajima and Baldridge^39^. They evaluated the effects of KT with tension and without tension on vertical jump height on fifty-two subjects (28 men and 24 women) randomized into 2 groups: 1) KT with tension, and 2) KT without tension. The tape was applied at the gastrocnemius and soleus, for muscle activation. No significant differences were found in the measurements of vertical jump height. According to Nakajima and Baldridge^39^, one possible explanation for the present findings is that the tactile input from the KT is not strong enough to increase muscle power to influence vertical jump height. This is in line with Petschnig et al.^21^, which found that the height of the vertical jump was attributed to the strength of the knee extensor muscles. Thus, it is possible to assume that KT did not produced increases in knee extensors muscle strength and, consequently, did not influence the jump height. Similarly, Huang et al.^12^ evaluated the performance of vertical jump in thirty-one healthy individuals (19 men and 12 women). They used an application similar to the Nakajima’ study, in which the tape was applied for the activation of the triceps surae, however, the vertical jump was performed with both limbs. They found a significant increase in vertical ground reaction force and EMG activity of the medial gastrocnemius during the jumping task with the KT. For jump height, no significant effects were found. The comparisons of our study with Huang and colleagues^12^ must be carefully done, considering that in the present study the tape was applied on the rectus femoris while Huang et al.^12^ applied it on the triceps surae. In addition, a squat jump may have influenced the difference between studies. Probably, the neuronal input of KT is not sufficient to increase hop test performance^23^ and muscle strength^44^.

Nevertheless, it is possible to assume that the elastic property of the tape allows free joint motion and could offer a mechanism to increase joint loading and muscle activity^12^ and may explain the moderate effect size found for the KG`s rate of force development at T0 and T24. However, an interesting finding was that the CG also presented an increase of RFD with moderate effect size at T0 and large effect size at T48. This is an unexpected finding that raises an important question regarding the different applications of KT and tape tension. The absence of performance increments of hop tests and vertical jump in the present study may be explained by the tension applied to the tape (40% of the rest length). This is an important feature of clinical practice, considering that tape tension is a key element of the KT method. The guidelines of the method claim that a tension of approximately 25-35% must be applied when the aim is to stimulate a muscle. However, the literature reported a broad range of tension and no standardized application procedures have been used. Unlike previous studies, the present study adopted an equation in order to minimize the subjectivity around tape tension; however, this issue is warranted in future studies.

One limitation of the present study was the use of KT in one muscle group only, during a multi-joint task. Thus, future studies should consider the use of KT with multiple applications (e.g. quadriceps femoris and triceps surae). Another limitation was the lack of assessor’s blinding, which should be observed in clinical trials with the KT.

## Conclusion

The present study demonstrated no immediate or prolonged effects of KT during the performance of the hop and vertical jump tests. Likewise, there were no significant differences between KT application with tension when compared to a condition without tension. Therefore, the KT method is not recommended when the objective is to improve the functional or proprioceptive performance of healthy, physically active individuals.
